# PINCH in the Cellular Stress Response to Tau-Hyperphosphorylation

**DOI:** 10.1371/journal.pone.0058232

**Published:** 2013-03-12

**Authors:** Ahmet Yunus Ozdemir, Inna Rom, Jane Kovalevich, William Yen, Radhika Adiga, Rajnish S. Dave, Dianne Langford

**Affiliations:** Temple University School of Medicine, Department of Neuroscience, Philadelphia, Pennsylvania, United States of America; Case Western Reserve University, United States of America

## Abstract

Particularly interesting new cysteine- histidine- rich protein (PINCH) is an adaptor protein that our data have shown is required for neurite extension under stressful conditions. Our previous studies also report that PINCH is recalled by neurons showing decreased levels of synaptodendritic signaling proteins such as MAP2 or synaptophysin in the brains of human immunodeficiency virus (HIV) patients. The current study addressed potential role(s) for PINCH in neurodegenerative diseases. Mass spectrometry predicted the interaction of PINCH with Tau and with members of the heat shock response. Our *in vitro* data confirmed that PINCH binds to hyperphosphorylated (hp) Tau and to E3 ubiquitin ligase, carboxy-terminus of heat shock-70 interacting protein. Silencing PINCH prior to induction of hp-Tau resulted in more efficient clearance of accumulating hp-Tau, suggesting that PINCH may play a role in stabilizing hp-Tau. Accumulation of hp-Tau is implicated in more than 20 neuropathological diseases including Alzheimer's disease (AD), frontotemporal dementia (FTD), and human immunodeficiency virus encephalitis (HIVE). Analyses of brain tissues from HIVE, AD and FTD patients showed that PINCH is increased and binds to hp-Tau. These studies address a new mechanism by which AD and HIV may intersect and identify PINCH as a contributing factor to the accumulation of hyperphosphorylated Tau.

## Introduction

Limited regenerative capacity in adults makes neurons in the human CNS particularly vulnerable to stress. The heat shock response (HSR) machinery is one of the cell's first lines of defense against misfolded or abnormally accumulating proteins. Functions of the HSR include surveillance of proteins post-translationally and after cellular insult or stress. These stressors include diverse events such as chronic traumatic encephalopathy injury [1], Alzheimer's disease (AD) [2], HIV encephalitis (HIVE) [3], [4], and frontotemporal dementia (FTD) [5] and can manifest, in part, as accumulation of hyperphosphorylated Tau (hp-Tau). Interestingly, in subacute sclerosing panencephalitis (SSPE), the formation of neurofibrillary tangles (NFT) composed of hp-Tau [6] were associated with measles virus [7], suggesting that viral infection can also contribute to NFT formation. Aberrant proteins that cannot be repaired may be targeted to the ubiquitin-proteasome system (UPS) for degradation. The HSR works with the UPS for the recognition and clearance of aberrant proteins through the binding of a series of chaperone proteins and attachment of ubiquitin molecules to the client protein. Briefly, the heat shock protein 90 (Hsp90) has inherent ATPase activity, and studies suggest that Hsp90 may either 1) initiate the repair response for refolding and/or dephosphorylation, or 2) target clients including hp-Tau for UPS degradation by recruiting the E3 ubiquitin ligase, carboxy-terminus of heat shock-70 interacting protein (CHIP) [8]. This recruitment is believed to be through CHIP's interaction with Hsp70/40 [9], [10]. Studies in the brains of AD patients show that the degree of CHIP expression is inversely proportional to the levels of aggregated Tau [11], [12]. Likewise, studies in mice deficient for CHIP show increased CNS accumulation of aberrant proteins and decreased proteasomal activity [13]. Recent studies also confirm important roles for Hsp70 and Hsp90 in controlling abnormal Tau accumulation [8], [14], [15], [16]. Consequent CHIP binding interferes with Hsp90-mediated refolding and facilitates ubiquitination of the client protein. However, if the cellular machinery fails to clear abnormal proteins, accumulation of aberrant proteins, such as hp-Tau may occur and lead to disease. Tau is ubiquitously expressed in the brain where it assembles and stabilizes microtubules in neuronal axons. Normally, the HSR complex sorts aberrant hp-Tau for either repair or degradation, as described above. Upon hyperphosphorylation, Tau dissociates from microtubules and may be redistributed to the cell body where it accumulates and forms fibrillary deposits consisting of paired helical filaments (PHF) to form NFT. Neurofibrillary tangles are composed in part of ubiquitinated hp-Tau and recent studies report that both the proteasomal and autophagosomal pathways are involved in hp-Tau degradation. However, controversy exists regarding the preferential degradation of specific forms of hp-Tau [9], [17].

Many cellular factors have been identified as contributors to the decision-making process of whether a client protein will be repaired or degraded. In this context, we have discovered that Particularly Interesting New Cysteine-Histidine-rich (PINCH) protein is upregulated in the neurons of patients with CNS disorders that have a Tau misfolding component, such as HIVE and AD. During development, PINCH is required to maintain neuronal polarity and synaptodendritic communication [18]. In health and during normal ageing, PINCH expression in the adult CNS is nearly undetectable in most cases with significantly lower levels in normal cases than in disease [19]. We first discovered that in the brains of HIV-infected patients, PINCH expression was recalled by neurons showing decreased levels of the synaptodendritic signaling proteins MAP2 and synaptophysin [19], [20]. Our more recent studies show that PINCH is required for neurite extension in neurons challenged with TNF-α [20], a mediator of synaptic dysfunction in the pathogenesis of many CNS diseases including AD and HIVE. In this context, studies show that in the frontal cortex of HIVE patients, hp-Tau levels are also increased [3]–[4].

PINCH is a highly conserved protein composed of 5 double zinc-finger domains and has no reported catalytic activity [21]. PINCH is a key component in the formation of multi-protein complexes and facilitates cell spreading, migration and survival. Although mammals have two PINCH proteins, 1 and 2, PINCH1 knockout is embryonic lethal, whereas PINCH2 knockout has no apparent consequences [22], [23]. PINCH1 and 2 share approximately 82% homology, are encoded by different genes and in some studies, a partial compensation of PINCH1 and 2 for one another is reported in later stages of development [24]. One of PINCH's most studied binding partners, integrin linked kinase (ILK), has been shown to interfere with GSK3-β-mediated Tau phosphorylation and, in some systems, ILK's activity is dependent on its binding to PINCH [25]. Our data suggest that PINCH1 acts as a key component in the CHIP/hp-Tau complex and is important for neuronal response to stressors that activate the HSR.

## Materials and Methods

### Neurons

*SH-SY5Y* (ATCC) cells, a noradrenergic subclone of the SK-N-SY neuroblastoma cell line were cultured in DMEM with 10% fetal bovine serum and 200 nM glutamine (Invitrogen, Carlsbad, CA, USA). Neurons were exposed to cell-free JR-CSF HIV (equivalent of 100 pg p24/cell, NIH AIDS Research and Reagent Program), normal media, or conditioned media from PBMCs infected with JR-FL (the equivalent of ≥100 p24 pg/ml), as determined by the HIV-1 p24 ELISA (ABL, Inc., Rockville, MD, USA). TNF-α levels in supernatants were determined using the human TNF ELISA kit II (BD Biosciences, San Jose, CA, USA). To induce hp-Tau, neurons were exposed to either TNF-α (100 ng/ml, Sigma, St. Louis, MO, USA) for 72 h or to okadaic acid (OA) (50 nM, Enzo Life Sciences, Farmingdale, NY, USA) for 2 h [26], [27], [28].

### Extraction of soluble cellular proteins

Neurons were harvested, homogenized in RIPA buffer (50 mM Tris (pH 8.0), 150 mM NaCl, 1% NP40, 5 mM EDTA, 0.5% sodium deoxycholate, 0.1% sodium dodecyl sulfate) and soluble fractions were separated by centrifugation at 14,000 rpm for 5 min at 4°C. Protein concentrations were determined by the Bradford assay (BioRad, Hercules, CA, USA) following the manufacturer's protocol.

### Western Analyses

Equal amounts of protein per well were loaded into 4–12% Bis-Tris pre-cast mini or midi-gels (Invitrogen), separated by electrophoresis and transferred onto nitrocellulose membranes (Bio-Rad). Membranes were blocked in 5% nonfat milk in Tris-buffered saline with Tween-20 (TBST) for 30 minutes before incubation with primary antibodies.

### Antibodies

Primary antibodies were used at 1∶1,000 for Western analyses unless otherwise indicated. PINCH1, (BD, Rockville, MD, USA), PINCH2 [22], Hsp90 (Abcam, Cambridge, MA), Hsp70 (1∶5,000) (Abcam), CHIP (1∶500) (Abcam), GAPDH (1∶5,000) (SCBT, Santa Cruz, CA, USA), or Grb-2 (Cell Signaling, Danvers, MA, USA). Briefly, anti-Tau antibodies from Thermo-Fischer (Pittsburgh, PA) included: HT7 against human total Tau, AT8 (detects phospho-S202, -T205, -S199, -S208) and AT100 (detects phospho-S121, -T214) against PHF-Tau. Other hp-Tau specific antibodies included phospho-S262 and -S396 (Abcam). Other antibodies included anti-ILK (SCBT), anti-Nck2 (SCBT), and anti-MAP2 (Cell Signaling). Membranes were incubated for either 2 h at 23°C or overnight at 4°C, washed in 1X TBST, incubated with appropriate secondary anti-mouse or -rabbit antibodies (1∶10,000; Abcam) for 1 h at 23°C, and developed with ECL or ECL PRIME (Amersham Pharmacia Biotech, Piscataway, NJ, USA). Band intensities were calculated using *ImageJ* software [29] and normalized to loading controls, Grb-2 or GAPDH.

### Immunoprecipitation

For immunoprecipitations, neurons were lysed in RIPA lysis buffer and incubated for 30 min on ice. To remove serine, threonine and tyrosine phosphates, lysate was treated with 10,000 units of λ protein phosphatase (New England BioLabs, Ipswich, MA, USA) for 2 h at 30°C and processed as described. Two hundred-fifty µg of total protein were incubated with 2.5 µg of antibody overnight at 4°C with end-over- end rotation. After incubation, 20 µl of Protein-A bead slurry was added and samples were rotated end-over-end to mix for 4 h at 4°C. The beads with protein conjugates were washed 5 times with 500 µl of lysis buffer. After centrifugation and removal of supernatant, 50 µl of 1X Laemmli sample buffer (Bio-Rad) was added to bead pellet. Samples were heated to 100°C for 5 min, centrifuged and the proteins were analyzed by Western blotting.

### Mass Spectrometry

Neurons were lysed and proteins were immunoprecipitated with anti-PINCH antibody and separated by standard SDS-polyacrylamide gel electrophoresis as described above. The gel was stained with Coomassie brilliant blue and visible bands were excised for analyses. Standard mass spectrometric peptide mapping was conducted and analyzed using MASCOT software (Matrix Science) [30] at the University of Pennsylvania Proteomics and Systems Biology Core facility (Philadelphia, PA).

### shRNA Infections

Mission® shRNA bacterial glycerol stock was expanded in 293T cells with packaging plasmids, PLP1, PLP2 and PLP VSVG, according to manufacturers' instructions (Sigma). Cell-free media was collected and neurons were infected with PINCH1 and PINCH2 shRNA, control pLKO.1-puro plasmid, or pCMV-GFP for infection efficiency. Neurons were harvested 72 h post-infection and Western analyses were conducted on cell lysate. Two different target shRNA plasmids (pLKO.1-puro) for human PINCH1 (NM_004987) were used: 1) shP1RNA1 (TRCN0000059038) and 2) shP1RNA3 (TRCN0000059040) (Sigma). For human PINCH2 (NM_017980), two different target shRNA plasmids were also used: 1) shP2RNA1 (TRCN0000244780) and 2) shP2RNA2 (TRCN0000244781). shRNA sequences are listed in Table 1.

**Table 1:** shRNA sequence data for human PINCH1 and 2.

human PINCH1	NM_004987	sequence
shP1RNA1	TRCN0000059038	CCGGCCCTTATCCATTTGTTGACATCTCGAGATGTCAACAAATGGATAAGGGTTTTTG
shP1RNA3	TRCN0000059040	CCGGGCTGAGACCTTAGGAAGGAAACTCGAGTTTCCTTCCTAAGGTCTCAGCTTTTTG
human PINCH2	NM_017980	
shP2RNA1	TRCN0000244780	CCGGCTGCGAACACGACTTCCAAATCTCGAGATTTGGAAGTCGTGTTCGCAGTTTTTG
shP2RNA2	TRCN0000244781	CCGGTCACCCTGAAGAACAAGTTTGCTCGAGCAAACTTGTTCTTCAGGGTGATTTTTG

### PINCH1 mutagenesis

Five LIMS-specific deletion mutants were generated using a PCR-based deletion strategy from full length PINCH1 on the pCMV6 plasmid vector containing Myc-DDK fusion tags to allow for capture and detection of exogenously expressed PINCH using anti-FLAG or -Myc antibodies (OriGene, Rockville, MD). Each LIM domain is 50–60 amino acids in length. To generate large deletion mutants from a plasmid DNA template, we utilized partially phosphorothioate-modified PCR primers and T7 gene 6 exonuclease digestion to introduce sticky ends at the ends of the PCR products, as previously described [31]. Table 2 lists the primers used to generate the five LIM-specific deletion mutants with asterisks representing positions of the phosphorothioates.

**Table 2:** PINCH deletion mutants and sequences.

Designation	Region Deleted	Forward primer	Reverse primer	Sequencing primer
LIM1	Δ[9–62aa] Δ[25–186 bp]	PINCH187F	PINCH24R	CMV Forward
LIM2	Δ[70–121aa] Δ[208–363 bp]	PINCH364F	PINCH207R	CMV Forward
LIM3	Δ[134–184aa] Δ[400–552 bp]	PINCH553F	PINCH399R	HPINCH300F
LIM4	Δ[192–243aa] Δ[574–729 bp]	PINCH730F	PINCH573R	HPINCH300F
LIM5	Δ[251–303aa] Δ[751–909 bp]	PINCH910F	PINCH750R	HPINCH600F
LIM1: HPINCH187F 5′-AGCGCCTTTCAGA*T*G*C*TCTTTGCC-3′				
LIM1: HPINCH24R 5′-CTGAAAGGCGCTG*G*C*C*AGGGCGTT-3′				
LIM2: HPINCH364F 5′-GCCCCTCATAATC*G*T*G*AGAAAGCC-3′				
LIM2: HPINCH207R 5′-ATTATGAGGGGCA*A*A*G*AGCATCTG-3′				
LIM3: HPINCH553F 5′-AAATACCATGATA*A*A*A*TGGGGGTC-3′				
LIM3: HPINCH399R 5′-ATCATGGTATTTC*C*C*A*AGGCCTCT-3′				
LIM4: HPINCH730F 5′-GTCCCCTATAACC*A*G*C*TATTTGGT-3′				
LIM4: HPINCH573R 5′-GTTATAGGGGACC*C*C*C*ATTTTATC-3′				
LIM5: HPINCH910F 5′-GGTGATTATGAGA*A*A*T*TTCCATTG-3′				
LIM5: HPINCH750R 5′-CTCATAATCACCA*A*A*T*AGCTGGTT-3′				
Sequencing Primers:				
HPINCH300F 5′-CCAGGAAGTTCTGGCAGATAT-3′				
HPINCH600F 5′-CGAAGGGCGCGTGGTGAACGCTAT-3′				
CMV Forward 5′-CGCAAATGGGCGGTAGGCGTG-3′				

### Human Brain Tissue

Human brain tissue was obtained from the Alzheimer's Disease Research Center (ADRC), (La Jolla, CA), the California NeuroAIDS Tissue Consortium (CNTN) (San Diego, CA), National NeuroAIDS Tissue Consortium (NNTC), the University of Pennsylvania Center for Neurodegenerative Disease Research (CNDR), Alzheimer's Disease Core Center (ADCC) and Udall Center for Parkinson's Research in accordance with Temple University Human Subjects Protections and the Institutional Review Board.

### Mouse Brain Tissue

Tau transgenic (Tau-Tg) mice, B6; C3-Tg (Prpn-MAPT*P301S), that express mutant human microtubule associated protein Tau under the direction of the mouse prion protein promoter and wild type mice were obtained from the Jackson Laboratory (Bar Harbor, ME, USA). Mice were heavily anesthetized with 5% isoflurane and decapitated in accordance with Temple University IACUC guidelines. Brains were removed and placed into ice-cold PBS. The left hemispheres from the wild-type and Tau-Tg mice were dissected into the following regions: *anterior frontal cortex* (Ant-FC) (1.32 mm anterior of Bregma), temporal lobe (∼30 mg of tissue taken from *ventral-lateral posterior frontal cortex* (V-L-post-FC) section), *posterior frontal cortex* (post-FC) (1.32 mm anterior of Bregma to 2.92 mm posterior of Bregma), and *cerebellum* (CB) (2.92 mm posterior of Bregma including only the cerebellum) and each region was processed for protein analysis and stored at −80°C. For immunohistochemical analyses, right hemispheres from wild type and Tau-Tg mice were fixed in 10% buffered formalin for 24 h and processed for immunolabeling by standard paraffin embedding and sectioning.

### Protein Extraction from Tissues

Proteins of different solubility were extracted from brain in buffers of increasing stringency, using a modified protocol [32]. Briefly, frozen brain tissue from gray matter of the frontal cortex was weighed and 100 mg was homogenized in 10 µl/mg RAB buffer (100 mM 2-(N-morpholino) ethanesulphonic acid (MES; pH 7.0), 1 mM EDTA, 0.5 mM MgSO_4_, 750 mM NaCl, 20 mM NaF, 1 mM Na_3_VO_4_ and complete protease inhibitors (Sigma)) with a plastic pestle in 1.5 mL tubes. The homogenate was passed through a 29G needle, incubated on ice for 30 min and centrifuged at 50,000×g for 20 min at 4°C. The RAB-soluble proteins in the supernatant were collected. The pellet was resuspended in 7.5 µl/mg RIPA buffer and centrifuged at 50,000×g for 20 min at 4°C. The supernatant containing RIPA-soluble proteins was collected. The pellet was resuspended in 7.5 µl/mg 70% formic acid (FA) in distilled water. The samples were incubated for 30 min on ice and centrifuged at 50,000×g for 20 min at 4°C. The supernatants containing FA-soluble proteins (also considered RIPA insoluble proteins) were collected. The FA fractions were dialyzed against PBS overnight at 4°C, and an equal volume of 50 mM Tris-HCl (pH 7.4) was added to each sample. Protein concentrations were determined by the Bradford assay and equal amounts of protein were loaded per well.

### Double Immunofluorescence Labeling and Deconvolution and Confocal Microscopy

Formalin-fixed, paraffin-embedded frontal cortex brain tissues from HIV, AD, and control patients were obtained from the CNTN and ADRC tissue repositories, respectively, and immunofluorescent labeling was conducted on serial sections. Four month-old male mice (Tau-Tg and wild type) were euthanized, brains were removed and one hemisphere was fixed in formalin for immunolabeling. Five or 40 µm serial sections from the formalin-fixed paraffin-embedded tissues were processed in citrate buffer for antigen retrieval and rehydrated through ethanol to water. Sections were blocked with normal human serum, incubated with the primary antibodies: anti-PINCH (1∶200) [19], anti-Tau AT8 (1∶200), anti-CHIP (1∶100), Hsp-70 (1∶200), and Hsp-90 (1∶200) overnight in a humidified chamber at room temperature, rinsed three times with PBS, then incubated with fluorescein isothiocyanate (FITC)-conjugated secondary antibody (1∶500) or Texas-red isothiocyanate (TRITC)-tagged secondary antibodies (1∶200) (Thermo-Scientific) for 2 h at room temperature in the dark. After washing with PBS, the sections were re-blocked and incubated overnight at room temperature in a humidified chamber with the second primary antibody. After washing, sections were incubated with the second secondary antibody for 1 h at room temperature in the dark. Finally, sections were cover-slipped with an aqueous based mounting media containing DAPI for nuclear labeling (Vector Laboratories), visualized with a Nikon ultraviolet inverted microscope, and processed with deconvolution software (Slidebook 4.0, Intelligent Imaging, Denver, CO). Deconvolution was performed using SlideBook4 software, allowing acquisition of multiple 0.2 mm thick digital sections and 3-D reconstruction of the image. Confocal microscopy was conducted on 40 µm sections using the Leica EL6000, with LAF AS software (Leica Microsystems, Buffalo Grove, IL, USA).

### Statistics

Data were generated from at least three independent experiments. Statistical analyses were conducted using the Prism 4 GraphPad software program (GraphPad Software, Inc.). Data were analyzed either by one-way ANOVA with post-hoc analyses or one-tailed paired student's T-test where appropriate and results were considered significant if p≤0.05.

## Results

### Induction of PINCH, Tau hyperphosphorylation and the Heat Shock Response *in vitro*

We reported increased PINCH protein levels in HIV-infected patients' brains in neurons showing a loss of synaptodendritic proteins and in neurons exposed *in vitro* to TNF-α or to the HIV protein Tat [19], [20]. Apart from these observations, the role(s) that PINCH may play in neuronal signaling during CNS disease have not been addressed. To investigate the potential function of PINCH in neurons during CNS disease and to identify new PINCH protein-protein interactions, we conducted mass spectrometric analyses on neurons exposed to TNF-α *in vitro*. After exposure to TNF-α for 72 h and immunoprecipitation with anti-PINCH antibody, bands from the eluted fractions were analyzed. Along with the expected hits, such as ILK and Nck-2, other top hits included microtubule-associated binding proteins and heat shock protein response (HSR)-related proteins that are involved in the clearance of abnormal cellular proteins (data not shown).

**Figure 1:**
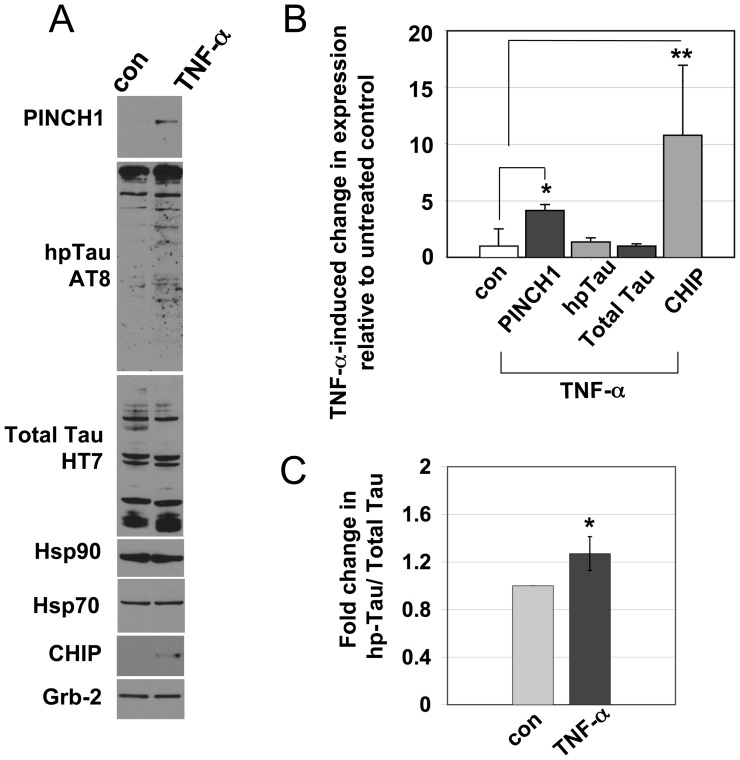
Exposure of neurons to TNF-α results in increased levels of PINCH, hp-Tau and CHIP. Representative Western blot of A) neurons exposed to TNF-α; B) graphic representation of fold change of PINCH1, hp-Tau, total Tau and CHIP levels in TNF-α treated neurons over control. Results are from 4 separate experiments and are expressed as fold change over control. * p<0.005, **p<0.001 by one-way ANOVA with Tukey-Kramer post-hoc analyses. Grb-2 was used as the loading control. C) Fold change in hp-Tau/Total Tau * p = 0.0293 by one-tailed paired T-test.

**Figure 2:**
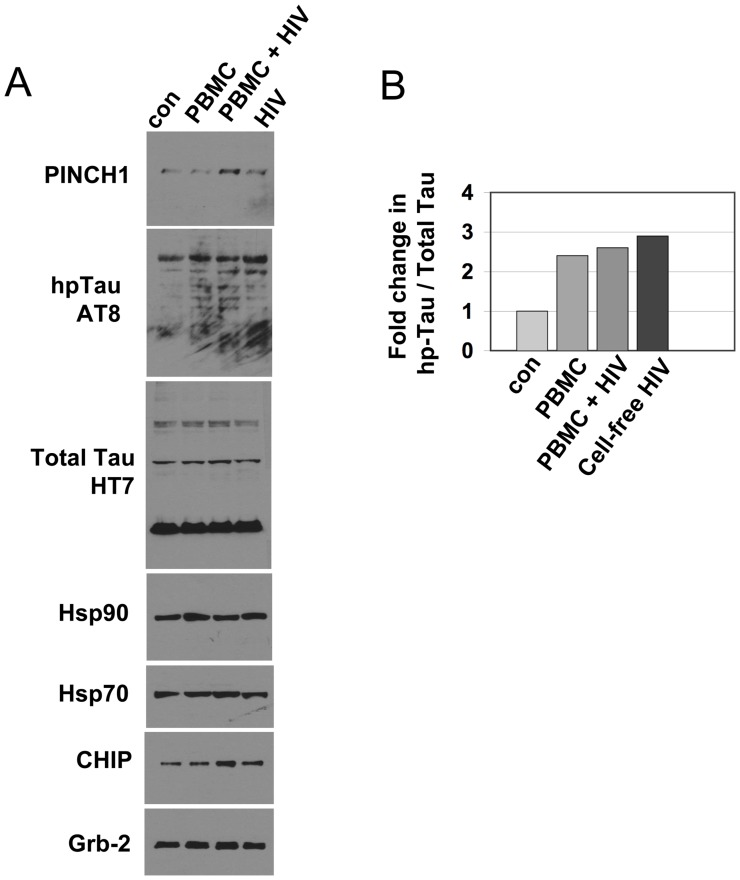
Exposure of neurons to supernatant from HIV-infected PBMCs results in increased PINCH, hp-Tau and CHIP. A) Representative Western blot showing levels of PINCH1, hpTau, total Tau, Hsp90, Hsp70 and CHIP from neurons grown in media without PBMCs (con), neurons exposed to supernatant from control PBMCs without HIV infection (PBMC), supernatant from PBMCs infected with HIV (PBMC+HIV), or neurons exposed to cell-free virus (HIV). Grb-2 was used as the loading control. B) Fold change in hpTau/Total Tau.

Neuronal response to HIV infection and AD involve heat shock proteins and changes in the microtubule-associated protein, Tau's phosphorylation state and its association with microtubules. To confirm the predicted involvement of HSR proteins, human neuronal cells were exposed to TNF-α, supernatant from HIV-infected PBMCs or cell-free virus and changes in levels of proteins were assessed. As previously reported, PINCH increased significantly in response to TNF-α [20] and these changes were accompanied by increased hp-Tau (*p<0.005) and CHIP (**p<0.001) protein levels (Figure 1A, B). Significantly more Tau was hyperphosphorylated in the TNF-α treated neurons (p = 0.0293) compared to the untreated neurons (Fig. 1C). No changes were observed in levels of Hsp90 or Hsp70 (Figure 1A). Neurons exposed to media from uninfected PBMCs showed low levels of PINCH and hp-Tau, whereas, exposure to supernatant from HIV-infected PBMCs increased PINCH, hp-Tau and CHIP (Figure 2A). As determined by ELISA, TNF-α levels in media from HIV-infected PBMCs ranged from 300–400 pg/ml at 4 days post- infection, ∼100 pg/ml in uninfected PBMC media, and was undetectable in media from cell-free virus or control media. Since neurons *in vitro* were exposed to 50–100 ng/ml TNF-α, it is likely that factors in addition to TNF-α are involved in PINCH induction. Increased hp-Tau was observed in neurons exposed to media from uninfected PBMCs, HIV-infected PBMCs and media containing cell-free HIV (Figure 2A, B). Given that cell-free virus did not induce PINCH expression, our results suggest different mechanisms influence the generation of hp-Tau and PINCH in this scenario.

### PINCH binds to Tau

**Figure 3:**
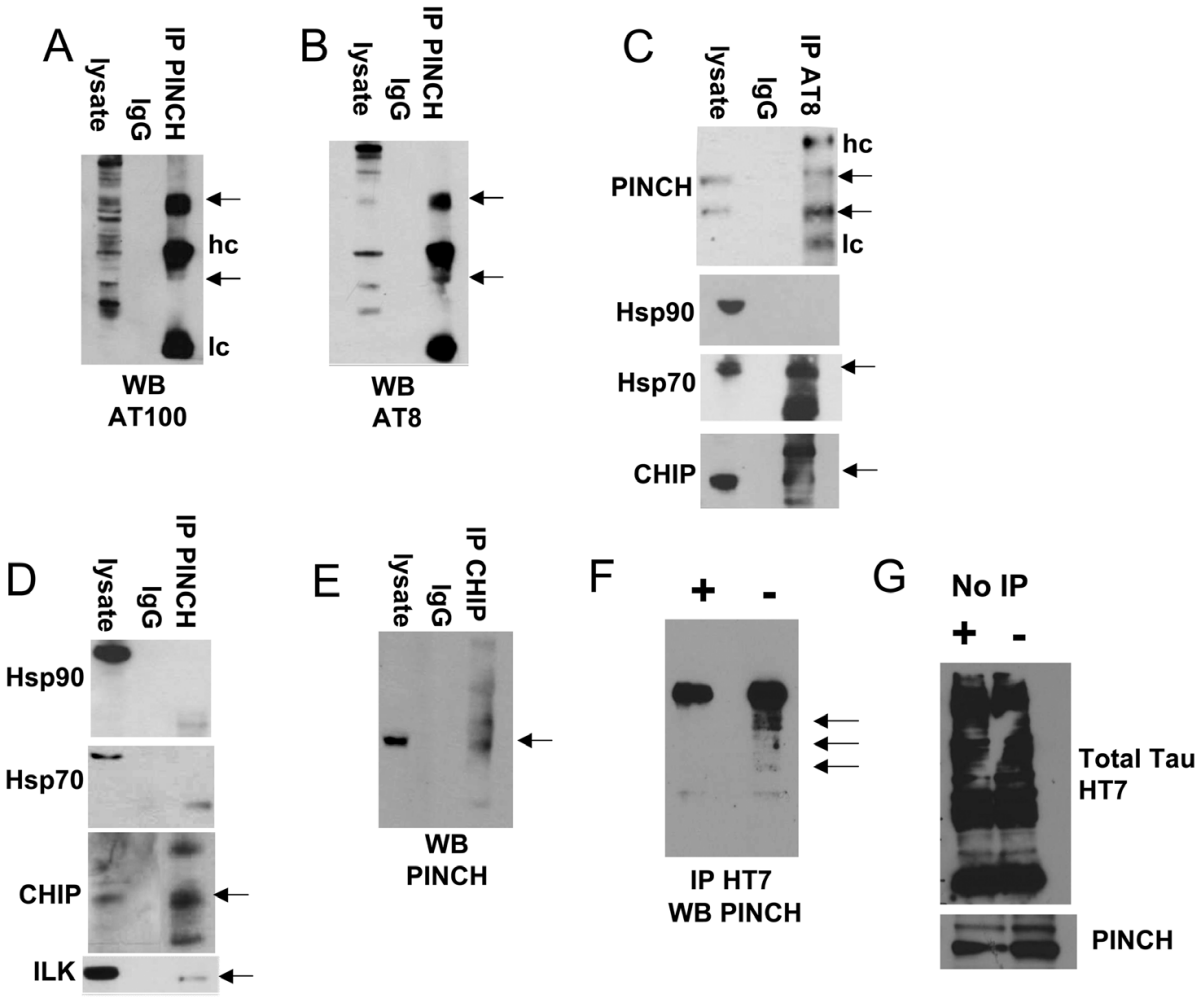
Reciprocal immunoprecipitations indicate PINCH interacts with hp-Tau and CHIP. Neuronal lysate was immunoprecipitated with anti PINCH and reacted with antibodies against hp-Tau A) AT100 and B) AT8. C) Neuronal lysate was immunoprecipitated with anti-AT8 antibody and reacted with anti-PINCH, anti-Hsp90, anti-Hsp70 and CHIP. D) Neuronal lysate was immunoprecipitated with anti-PINCH antibody and reacted with anti-Hsp90, anti-Hsp70, anti-CHIP and anti-ILK. E) Reciprocal IP with anti-CHIP and reaction with anti-PINCH. F) Neuronal lysate was treated with (+) or without (−) phosphatase and immunoprecipitated with anti-HT7 antibody against total Tau and reacted with anti-PINCH antibody. G) The same neuronal lysate with (+) and without (−) phosphatase was immunoblotted with anti-HT7 and anti-PINCH antibodies without immunoprecipitation. Arrows indicate immunoreactive bands. Hc, heavy chain; lc, light chain; No IP (lysate), beads only (IgG); immunoprecipitation (IP).

To confirm mass spectrometric analyses results, immunoprecipitation experiments were conducted with lysate from neurons exposed to TNF-α. Immunoprecipitation of neuronal lysate with anti-PINCH antibody and Western analyses with anti-Tau (AT100) (Figure 3A, arrows) and with AT8 (Figure 3B, arrows) indicated multiple hp-Tau immunoreactive bands phosphorylated at residues associated with PHF formation. Tau immunoreactive bands are detected both above and below the heavy chain (arrows). It is unclear whether Tau-immunoreactive bands exist corresponding to ∼50 kDa due to heavy chain cross-reactivity. Immunoprecipitation with the anti-Tau antibody (AT8) revealed two PINCH immunoreactive bands at approximately 37 kDa and 42 kDa, confirming the interaction of hp-Tau with PINCH (Figure 3C, arrows). PINCH immunoreactive bands corresponding to 37 and 42 kDa are reminiscent of those detected in patient tissues. In support of previous studies [33], our results showed hp-Tau interaction with Hsp70 and CHIP (Figure 3C, arrows). However, our data did not detect hp-Tau interaction with Hsp90 (Figure 3C). Of note however, the interactions of client proteins with HSR factors are cyclic [34] and analyses at a given time may not capture all interactions that occur.

Immunoprecipitation experiments to assess binding dynamics of PINCH with heat shock proteins and with the ubiquitin E-3 ligase, CHIP, confirmed that PINCH and CHIP interact (Figure 3D, arrow), but did not support the interaction of PINCH with Hsp70 or Hsp90 (Figure 3D). As expected, binding between PINCH and ILK was observed (Figure 3D, arrow). Reciprocal immunoprecipitation with anti-CHIP confirmed the PINCH-CHIP interaction (Figure 3E, arrow). To assess if PINCH interacts with de-phosphorylated Tau, neuronal lysate was treated with λ protein phosphatase, proteins were immunoprecipitated with anti-Total Tau antibody and reacted with anti-PINCH antibody (Figure 3F). No immunoreactive bands were detected in the de-phosphorylated sample; whereas, multiple PINCH immunoreactive bands were observed in the sample without phosphatase (3F, arrows). Western analyses of lysate without immunoprecipitation, with and without phosphatase indicated similar levels of Tau and PINCH protein (Figure 3G). Our data show that *in vitro*, in response to stressors such as TNF-α or other HIV-associated factors increased PINCH expression is accompanied by accumulation of hp-Tau and induction of the HSR. During induction of Tau phosphorylation, PINCH binds to hp-Tau and to the ubiquitin ligase CHIP.

### PINCH LIM domains 1 and 2 interact with Tau

**Figure 4:**
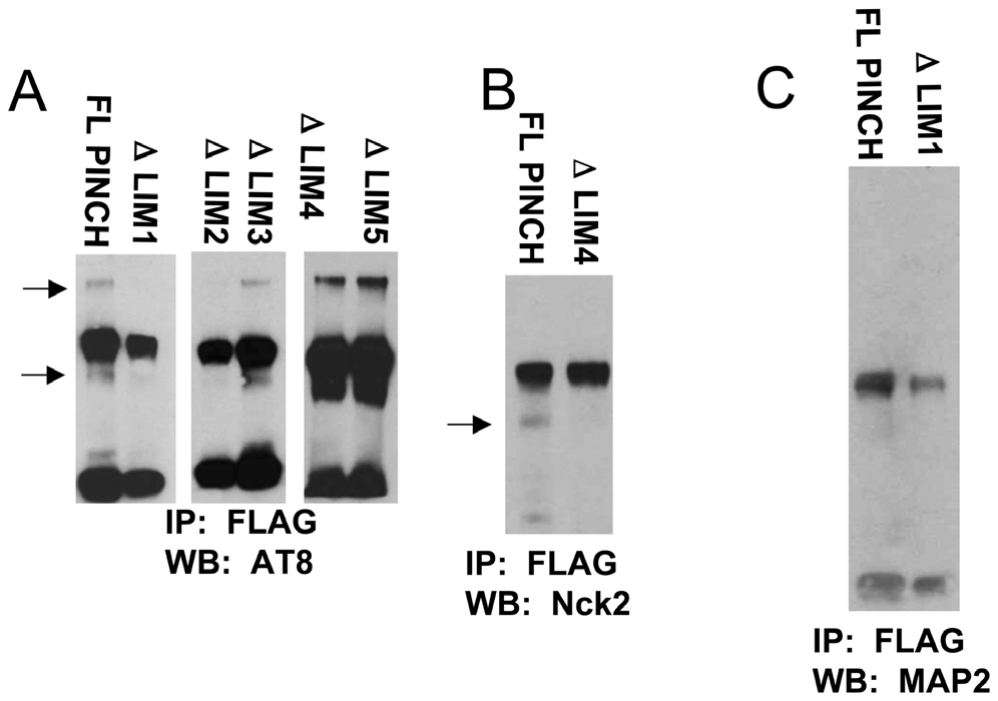
hp-Tau interacts with the first and second LIM domains of PINCH. A) LIMS-specific deletion mutations predict that LIM1 and 2 domains of PINCH1 bind to hp-Tau. Expression plasmids for mutants (ΔLIMS 1–5) and full-length PINCH each with a FLAG-tag were generated via a PCR based strategy. Neuronal lysates were immunoprecipitated with anti-FLAG antibody and reacted with A) anti-hp-Tau (AT8), B) Nck-2 or E) MAP2 antibodies. Arrows indicate immunoreactive bands. FL, full length; ΔLIM, delta LIM indicates the LIM domain that was deleted; FLAG, epitope N-DYKDDDDK-C tag; Nck2, cytoplasmic adaptor protein that interacts with PINCH LIM4; MAP2, microtubule associated protein-2.

To determine which region(s) of PINCH interact with Tau, five LIMS-specific deletion mutants were generated, each lacking one of the five LIM domains. Neurons were transfected with plasmids expressing full-length PINCH, or each of the five deletion mutants: ΔLIMS 1, 2, 3, 4 or 5. Lysates were immunoprecipitated with anti-FLAG antibody to capture only exogenously expressed PINCH and reacted with anti-Tau antibody. In the lysate from full-length PINCH expression, two bands are visualized at approximately 80 and 50 kDa (Figure 4A, arrows). No anti-Tau immunoreactive bands are present in the ΔLIM1 expressing lane and only a faint band is detected in the ΔLIM2 lane, suggesting that PINCH LIMS 1 and 2 are important for Tau interaction. Lysates from ΔLIMS 3, 4, 5 show clear immunoreactive bands with Tau indicating that these LIM domains are not involved in Tau interaction. As described by other studies, Nck2 interacts with full-length PINCH [35] and interaction is not observed in the ΔLIM4 mutant (Figure 4B) supporting the specificity of our LIMS mutants. Neither full-length PINCH nor ΔLIM1 interact with MAP2 (Figure 4C), lending support for PINCH's binding specificity for Tau.

### Biochemical signaling events in the PINCH/Tau HSR

**Figure 5:**
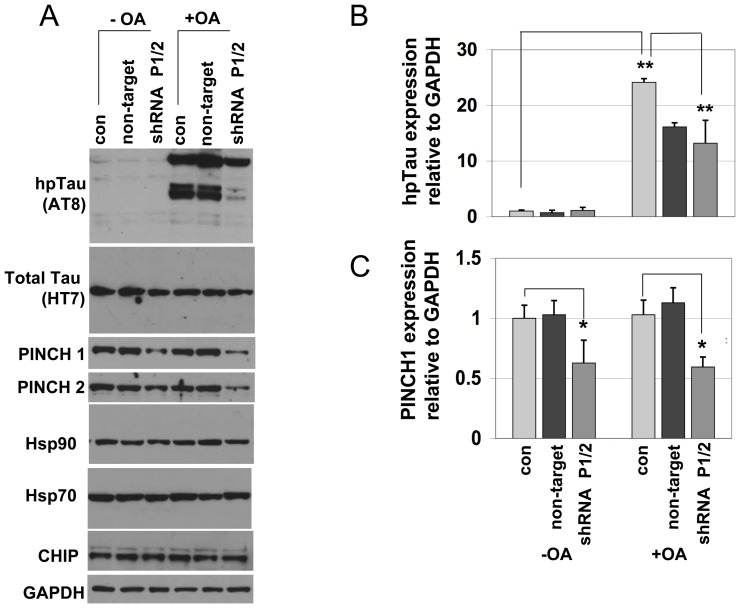
Silencing PINCH during hp-Tau induction results in less hp-Tau accumulation. A) Representative Western blots of neurons uninfected (con), infected with shRNA against PINCH1 and 2 (shRNA P1/2) or non-target control shRNA with and without okadaic acid (OA) treatment. B) Quantification of protein expression levels of hp-Tau (AT8), and C) PINCH relative to loading control (GAPDH). No changes were detected in Hsp90, Hsp70 or CHIP. Results are from 4 separate experiments and are expressed as fold change over uninfected neurons (con). * p<0.005, **p<0.001 by one-way ANOVA with Tukey-Kramer post-hoc analyses.

To address the potential significance of PINCH in neurons during conditions that lead to hp-Tau accumulation, PINCH was knocked down by shRNA prior to induction of hp-Tau (Figure 5A, C). As determined by cyclohexamide studies, the half-life of PINCH protein is approximately 72 h (data not shown); therefore, neurons were infected with PINCH1 and PINCH2 shRNA for 72 h prior to stress. Okadaic acid induces hyperphosphorylation of Tau in neurons after 2 h of exposure [36]. In our hands, 50 nm OA significantly increased levels of hp-Tau (Figure 5A). Silencing PINCH had no effect on levels of hp-Tau in neurons not exposed to OA. However, in neurons exposed to OA where PINCH was knocked down, significantly less hp-Tau was detected (Figure 4B, ** p<0.001). No significant changes were observed in levels of Hsp90, Hsp70 or CHIP (Figure 5A). These results suggest that PINCH promotes increased accumulation of OA-induced hp-Tau.

### PINCH, Tau and HSR factors in the brains of HIVE and AD patients

**Figure 6:**
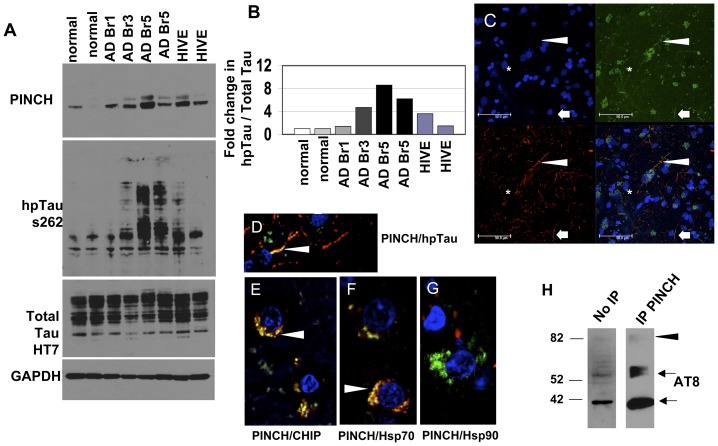
Expression levels of hp-Tau and PINCH protein in frontal cortex brain tissue from patients. A) Representative Western blot showing PINCH, soluble hp-Tau (s262) and total Tau (HT7) in age-matched controls (normal), AD Braak stages 1, 3, 5 (Br1, 3, 5), and HIV encephalitis (HIVE) patients. B) Graphic representation of percent total Tau that is hyperphosphorylated (hpTau/total Tau) in each case compared to control. C-G) Double immunofluorescence labeling of a frontal cortex tissue from a representative AD patient (Braak stage 3). C) from top left, panel shows DAPI for nuclei in blue; top right, PINCH in green; lower left, hp-Tau (s396) in red; lower right, co-localization of PINCH and hp-Tau. Arrowheads, arrows and asterisks indicate the same area in the tissue section. D) Double-immunolabeling of PINCH (green) and hp-Tau (red), co-localization, yellow; E) PINCH (green) and CHIP (red), co-localization (yellow); F) PINCH (green) and Hsp70 (red), co-localization (yellow); G) PINCH (green) and Hsp90 (red), no co-localization; H) Brain homogenate from a representative AD patient (Braak stage 3) immunoprecipitated with anti-PINCH antibody and reacted with anti-hp-Tau (AT8) antibody. Arrows, arrowheads show immunoreactive bands indicating PINCH-hp-Tau interaction.

Our *in vitro* data in neuroblastoma cells show that PINCH and hp-Tau interact during the HSR upon treatments that induce hyperphosphorylation of Tau. Thus, to assess the relevance of these data to human disease, we assessed levels of soluble hp-Tau and PINCH in post-mortem brain tissue from normal controls, AD and HIVE patients (Figure 6A). PINCH is detected as a doublet at approximately 37 and 42 kDa (reminiscent of bands in Figure 3C), and compared to control, PINCH levels are increased in AD and HIVE, with a trend towards increased levels in more severe AD (compare Braak stages 1, 3, 5) (Figure 6A, B). Total Tau levels are similar in all cases. Since our *in vitro* data also showed that PINCH interacts with heat shock factors, double immunofluorescence labeling of brain tissue from a representative AD patient (Braak stage 3) was conducted (Figure 6C–G). Confocal imaging suggested PINCH/hp-Tau co-localization (Figure 6C, arrows, arrowheads and asterisks). Higher magnification suggested PINCH co-localization with hp-Tau (Figure 6D, arrowhead), Hsp70 (6E, arrowhead) and CHIP (6F, arrowhead), but not with Hsp90 (6G). Immunoprecipitation of tissue lysate from an AD patient's brain supported the interaction of PINCH with hpTau as shown by two immunoreactive bands at approximately 55–60 kDa, 42 kDa (Figure 6H, arrows) and a weaker band at 82 kDa (Figure 5H, arrowhead).

**Figure 7:**
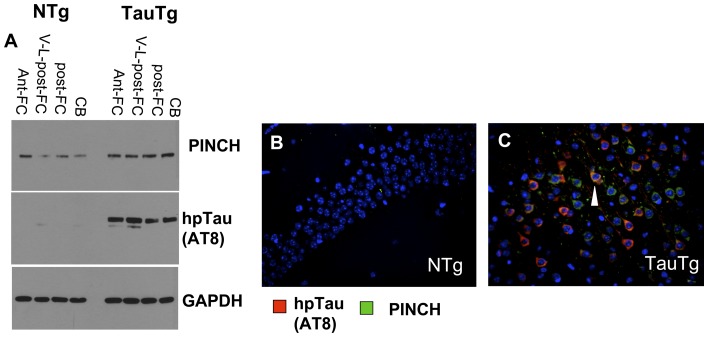
Expression levels of hp-Tau and PINCH in brain tissue from human Tau transgenic mouse. A) Western analyses of anterior frontal cortex (Ant-Fc), ventro-lateral posterior cortex (V-L-post-FC), posterior frontal cortex (post-FC), cerebellum (CB). B) Double immunofluorescence labeling of PINCH (green) and hp-Tau (red), co-localization (yellow) in hippocampal tissue from a wild-type mouse and the Tau-Tg mouse and respectively.

Further support for the interaction of hp-Tau and PINCH in diseases with a pathological component of hp-Tau was shown by significantly increased PINCH expression in the brain of the P301S human Tau transgenic mouse (Tau-Tg). Western analyses of different brain regions from the Tau-Tg mouse indicated increased soluble hp-Tau and PINCH in all regions (Figure 7A). Likewise, double-immunolabeling of hippocampal tissue indicated that in the P301S human Tau transgenic mouse, PINCH and hp-Tau were detected (Figure 7C); whereas, in the control, immunoreactivity was not detected for either protein (Figure, 7B). As observed in AD and HIVE patients' brains, PINCH and hp-Tau appeared to co-localize in the neurons of the Tau transgenic mouse (Figure 7C, arrowhead). Our results support numerous reports that hp-Tau levels increase in AD, HIVE and in the human-Tau transgenic mouse, and we now show that hp-Tau accumulation is accompanied by increased PINCH expression. These findings provide evidence for a possible role for PINCH in HIVE that intersects with neuropathological processes in AD.

### Changes in PINCH accompany decreased hp-Tau solubility

**Figure 8:**
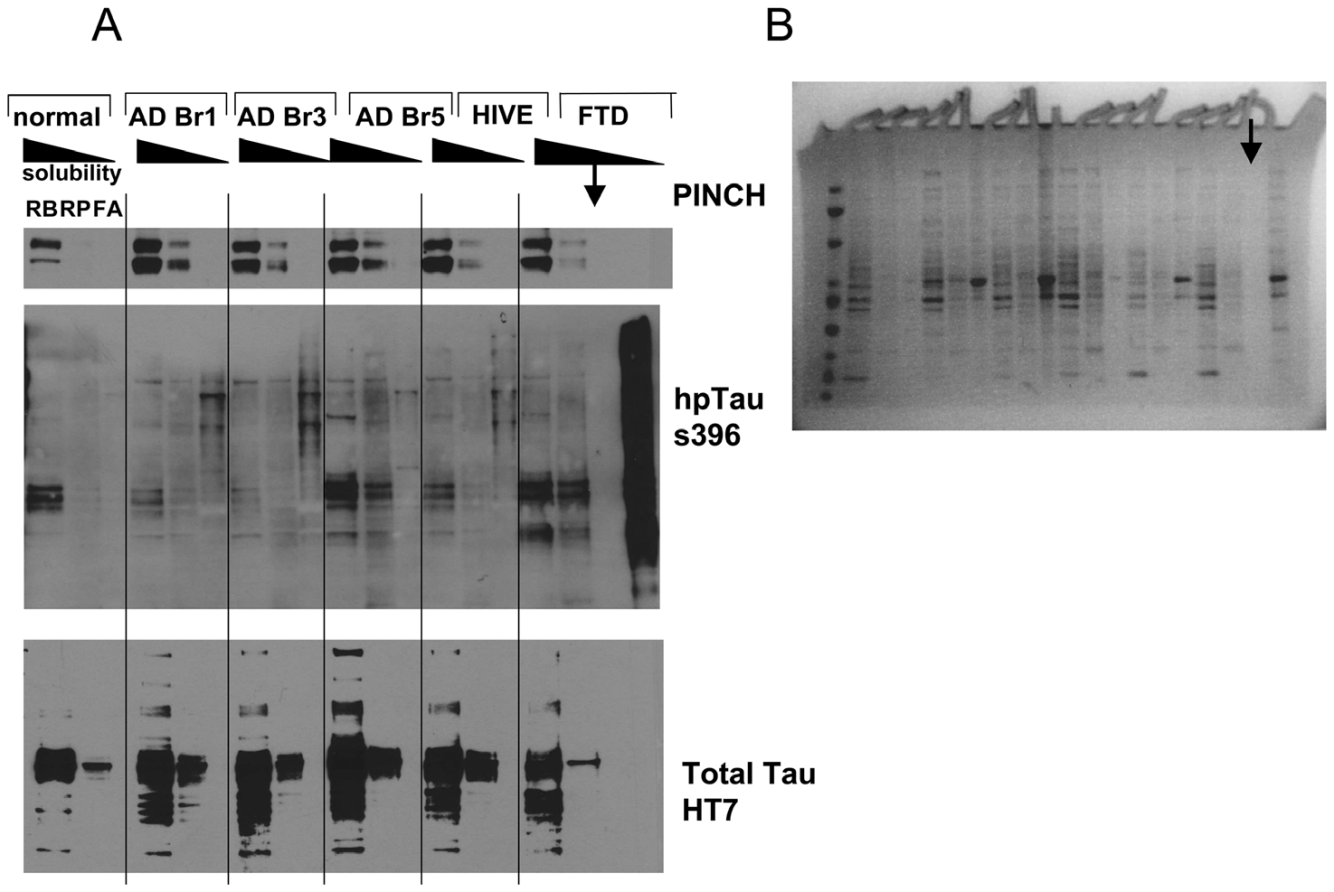
PINCH levels in relation to changes in Tau solubility. The proteins from frontal cortex homogenates from normal, different Braak stages (1, 3, 5) of Alzheimer's disease (AD), HIV encephalitis (HIVE), and frontotemporal dementia (FTD) patients were fractionated into RAB (most soluble, RB), RIPA (less soluble, RB) and formic acid (FA) (least soluble). Representative Western blots from A) normal control, AD, HIVE, and FTD showing levels of PINCH, hpTau (s396) and total Tau (HT7). B) Coomassie stained gel of the same cases indicating total protein in each fraction. Compared to the control, increased levels of hp-Tau and PINCH are observed in disease cases. Loss of Tau and PINCH solubility are apparent in AD, HIVE and FTD, as well. Arrow in the FTD case indicates a blank lane in the gel.

Upon hyperphosphorylation of Tau, the accumulation of PHF and NFT are accompanied by loss of hp-Tau solubility. To determine expression levels and solubility of PINCH during loss of Tau solubility in disease, brain tissues from normal control, AD, HIVE and FTD patients were processed to separate proteins into different fractions based on solubility, with the RAB (RB) fraction being soluble, RIPA (RP) less soluble and the formic acid fraction (FA), insoluble (Figure 8A). As expected, in brain tissue from a normal control case, low levels of PINCH were detected in the RAB preparation containing soluble proteins (Figure 8A). Low levels of hp-Tau were also detected in the RAB and RIPA fractions, with the majority of total Tau present in the highly soluble RAB fraction (Figure 8A), confirming that most of phosphorylated Tau in patients without tauopathy is highly soluble. These results show that in patients suffering from neurodegenerative diseases with a tauopathy component, PINCH is increased and binds to hp-Tau. In AD, HIVE and FTD cases, PINCH is also detected at approximately 37 and 42 kDa in both the RAB and RIPA fractions, indicting that PINCH loses solubility in disease. In the Braak stage 5 case a faint PINCH band at 37 kDa is visible in the FA fraction supporting that PINCH may lose solubility in more severe disease stages.

## Discussion

Tau-related neurodegeneration is likely due to a combination of loss of normal function and gain of toxic function, and multiple factors contribute to both processes. Many reports link hp-Tau accumulation with induction of the HSR and several heat shock factors have been proposed as potential therapeutic targets for more efficient clearance of abnormal Tau [14], [16], [37]. The biological significance of PINCH expression during hp-Tau accumulation appears to relate to the cell's ability to clear hp-Tau. Our data show that during OA-mediated induction of Tau hyperphosphorylation, the absence of PINCH protein results in lower levels of hp-Tau. Possible explanations for the detection of less hp-Tau include the generation of less hp-Tau in response to OA, more efficient clearance of hp-Tau, or both [2]. Given that in some systems PINCH is required for proper ILK functioning [38], [39], [40] and that ILK controls Tau phosphorylation via regulation of GSK-3β [25], it is possible that silencing PINCH may impact the phosphorylation levels of Tau. On the other hand, PINCH was shown to interact with the ubiquitin E3 ligase, CHIP, and with hp-Tau and changes in this dynamic in the absence of PINCH may result in increased ubiquitination of hp-Tau. Further studies are required to address both of these possibilities.

Members of the HSR machinery are not only important in chaperoning aberrant proteins to the UPS for degradation, but some factors, such as Hsp90, play critical roles in promoting stability of abnormal proteins and their accumulation inside the cell [16]. In fact, 2007 studies from Luo *et al.* describe Hsp90 as being a link between neurodegeneration and cancer [8]. In these studies, inhibition of Hsp90 with 17AAG and PU24FCI, in both *in vitro* and *in vivo* models of tauopathy, resulted in clearance of aggregated Tau [8]. In this context, PINCH has been shown to play an important role in cell survival in cancer by conferring resistance to apoptosis via the ERK-Bim pathway [41]. In fact, PINCH expression levels are significantly greater in many types of cancers, and in rectal cancer PINCH is an independent prognostic marker for worse outcome [41]–[44]. In our studies, levels of Hsp90 were not altered by TNF-α, OA treatment, or by silencing PINCH1 and 2 (Figures 1 and 4). Likewise, neither PINCH nor hp-Tau was observed to interact with Hsp90 upon immunoprecipitation and Western analyses (Figure 3). On the other hand, levels of CHIP were increased, and both PINCH and hp-Tau interacted with CHIP suggesting that the effects observed in this system may involve CHIP. However, the attachment of Hsp90 to its substrate is cyclic and its activity is ATP-dependent. Thus, our results may not be representative of all Hsp90 binding events that may have occurred and may not have captured changes in Hsp90 levels or its interactions with client proteins. Taken together, our data confirm an important role for PINCH in the HSR during accumulation of hp-Tau in AD, HIVE and FTD and in the human Tau transgenic mouse. Our *in vitro* data indicate that PINCH and Tau interact and that the first and second LIM domains of PINCH1 are important for this interaction. Silencing PINCH results in the detection of less hp-Tau in response to OA treatment, suggesting that PINCH may act to stabilize abnormal Tau or play a role in Tau hyperphosphorylation. Future confirmatory studies are required to elucidate the mechanism(s) through which PINCH is involved in hp-Tau formation and/or clearance.
